# Support services for rural women micro-entrepreneurs in Bangladesh: a sectoral comparison

**DOI:** 10.3389/fsoc.2025.1620857

**Published:** 2026-01-05

**Authors:** Jakia Begum, Kyoko Kusakabe, Takuji W. Tsusaka

**Affiliations:** 1 Gender and Development Studies, Asian Institute of Technology, Bangkok, Thailand; 2 Natural Resources Management, Asian Institute of Technology, Bangkok, Thailand

**Keywords:** women entrepreneur, micro-enterprise, support service, training program, sectoral comparison

## Abstract

**Introduction:**

Existing studies emphasize microcredit’s role in women’s entrepreneurship but often overlook other forms of support such as training and support services influencing sustainability and income growth. Furthermore, research exploring sectoral differences in the effects of these types of assistance has been insufficient. This paper analyzes the effects of training and support services on women’s micro-enterprises in rural Bangladesh and compares the differential effects in three sectors.

**Methods:**

A mixed-methods approach was used. A field survey was conducted with 170 randomly selected women entrepreneurs in the Tangail District: 63 in tailoring, 73 in embroidery, and 34 in basket-weaving. Descriptive and multiple regression analyses were used to identify support services that are important in each of the sectors. In-depth interviews were also carried out with 20 women entrepreneurs, and nine key informant interviews were organized with local officials and NGO representatives to gain insights into the implementation and roles of support services.

**Results:**

While training and support services enhance enterprise income, their effects vary by sector, likely due to differences in business operations, skill requirements, and resource dependence. Businesses in the tailoring sector benefit from skills-based training, while those in basket weaving gain from market access support. Sectoral differences were also found in how external factors affect financial performance, such as education, family support, and experience. These findings highlight a need for sector-specific strategies to empower rural women entrepreneurs.

## 1 Introduction

Alleviating poverty, enhancing household income, and reducing gender inequality by empowering women in decision-making processes in developing countries and providing support services and training for women in micro and small enterprises are fundamental strategies (Maksimov et al., 2017). A number of studies have demonstrated that targeted interventions significantly contribute to the sustainability and expansion of women-led businesses. For instance, microcredit initiatives have been extensively established as effective in fostering entrepreneurship and facilitating resource acquisition for small-scale enterprises (Singh and Dash, 2021). Similarly, training programs have been proven to advance entrepreneurial capabilities and business outcomes particularly those tailored to enhance technical and managerial skills (Yusuf and Ahmad, 2019). In addition, involvements in trade fairs, market access programs, and other initiatives facilitate networking opportunities and brand recognition which are essential for the expansion of micro-businesses (Bairagi and Ferdousi, 2020). These findings, therefore, highlight the diverse pathways through which support mechanisms can influence women entrepreneurs’ success (Bhuiyan and Ivlevs, 2019).

However, limited research has explored the effect of training and support services for women entrepreneurs on enterprise development by sector. While individual initiatives such as microcredit and skills training are well-documented, there remains a gap in understanding how income growth is influenced by the provision of support services and training across different business sectors.

This paper, therefore, investigates the type of training and support services that rural women entrepreneurs in tailoring, embroidery, and basket weaving received and the various effects these have on their businesses in Bangladesh.

## 2 Literature review

### 2.1 Challenges faced by women entrepreneurs

Women entrepreneurs experience numerous challenges that hinder their ability to establish, operate, and expand their businesses. As a result, female-owned businesses have lower profit, size, and productivity compared to male-owned enterprises as illustrated in some studies (Bardasi et al., 2021; Chaudhuri et al., 2020; Rosa and Sylla, 2018). The barriers can be classified into financial, socio-cultural, household, and informational barriers.

#### 2.1.1 Financial barriers

Financial constraints are one of the most significant barriers faced by women entrepreneurs. One reason is that restricted access to capital due to social, cultural, and institutional biases limits women’s ability to sustain or expand their businesses (Singh and Dash, 2021). Furthermore, patriarchal systems often hinder financial independence and decision-making power, preventing women from securing loans or credit (Cabeza-García et al., 2019). Women’s restricted access to financial resources is also primarily caused by lack of asset ownership, such as land, which often serves as a guarantee for loans (Group, W.B, 2022). In addition, women are constrained from owning or inheriting property in many developing regions because of legal and cultural practices further complicating the issue (World Bank, 2022; Mundial, 2022). In some countries, for example, legal restrictions are enforced requiring male co-signatories for women to obtain credits or sign contracts (International Finance Corporation, 2019).

Another important factor is the systemic barriers which include discrimination in financial institutions, high collateral requirements, and lack of tailored financial services. These impediments compel many women entrepreneurs to rely on informal lending or personal savings, which are generally inadequate for business growth (Rahman et al., 2022). Thus, restricting their access to formal credit, hindering business expansion, innovation, and long-term sustainability. Furthermore, women entrepreneurs often face additional hurdles, such as gender biases that question their business competence or reliability, further constraining their ability to secure financing (Atkins and Hale, 2018). However, some studies contest the presence of gender-based discrimination, suggesting that financial institutions have gender-neutral policies or even preferential terms for women borrowers due to government-led initiatives promoting women’s entrepreneurship (Johannes and Adze, 2020; Pedraza, 2021; Singh and Dash, 2021). For instance, programs such as subsidized loans and specialized credit lines are designed to address financial gaps for women entrepreneurs. While these initiatives can help reduce certain barriers, evidence indicates that their application is not consistent which resulted in many women being underserved, particularly those in rural areas where formal financial services are scarce. This conflicting evidence underscores the necessity for more comprehensive, accessible, and gender-sensitive financial frameworks to provide equitable support for all women entrepreneurs.

#### 2.1.2 Socio-cultural constraints

Another barrier is related to socio-cultural constraints. Women’s micro-enterprises face significant social and cultural challenges at various stages; start-up, survival, diversification, and growth (Akoh, 2020; Mbiti et al., 2015). This type of barriers result from the negative attitudes toward women in businesses, limited family support, and restrictions on travel and work hours (Kimuli et al., 2022) which hinder women from initiating or expanding their enterprises (Kimuli et al., 2022; Akoh, 2020). In some cultures, family responsibilities precede professional ambitions among women; thereby, limiting their ability to network or engage in entrepreneurial activities (Mbiti et al., 2015). These cultural expectations not only affect their confidence and self-perception but also lead to impediments in dealing with business environments (Goswami et al., 2019).

Despite these impediments, some countries like Indonesia, have made significant progress in achieving gender equality in entrepreneurship. Women-led enterprises are perceived as fundamental to the development of micro, small, and medium-scale enterprises (MSMEs), driving national economic growth and innovation (Anggadwita et al., 2017; Yesmin et al., 2024).

#### 2.1.3 Household responsibilities

Women’s household responsibilities are also a hindrance to their engagement in entrepreneurship. Women entrepreneurs often face the dual burden of managing household responsibilities alongside their business endeavors. They are generally expected to fulfill their roles as caregivers and homemakers based on social norms and expectations which significantly reduce the time and effort they can allocate for their businesses (Akoh, A., 2020). For instance, research conducted in Kenya and Malaysia underscored the effects of household responsibilities and business demand on women’s performance, particularly in micro and small enterprises (Maina, 2015; Karubi et al., 2014). Furthermore, the disproportionate share of household responsibilities generally faced by women perpetuates gender inequities, restricting women from participating successfully in the market (Campos and Gassier, 2017; Naldi et al., 2021). Several studies, however, revealed that women who receive support from their family can focus on and expand their business, and enhance their motivation and performance (Bagis et al., 2022; De Clercq et al., 2023).

#### 2.1.4 Limited access to knowledge, information, and networks

Finally, women’s limited access to knowledge, information, training, and social networks is another major barrier. Aragaw (2018) indicates that illiteracy, lack of business knowledge and managerial experience, and unavailability of training programs and technical support are the main causes of women’s difficulties. Therefore, women can be encouraged to participate in the economic life of their country by providing an encouraging environment for micro-enterprises alongside supportive organizational, legal, economic, and social incentives (Zainol et al., 2017). Notably, the absence of technical support and mentorship further constrains them from managing and advancing their enterprises (Abebe and Kegne, 2023).

Ultimately, targeted initiatives, such as financial literacy programs, mentorship opportunities and access to professional networks, will enable women to succeed in dealing with these barriers (Guragain and Doneys, 2022). As stated by Handaragama and Kusakabe (2021), providing improved support through education, social networks, targeted financing, and business assistance programs is necessary to enhance female entrepreneurship (Yesmin et al., 2024).

### 2.2 Support services for women’s micro-enterprise development

Support services are vital for empowering women entrepreneurs and enabling the sustainable growth of their micro-enterprises. The efficacy of financial, technical, managerial, and marketing support programs has been assessed through several evaluation studies, identifying their respective contributions and limitations. These studies provide a comprehensive understanding of how various types of support affect business performance across diverse sectors. (Bharti, 2019; Ekpe et al., 2015). An understanding of these sector-specific needs is crucial for designing more effective and targeted interventions that drive long-term success for women-led microenterprises.

Financial support, such as microcredit and other financial assistance programs has also been widely evaluated for its influence on women’s enterprises. Khandker and Samad (2014) and Banerjee et al. (2015) demonstrate positive effects of financial support on income generation and business growth as women entrepreneurs are provided with the necessary capital to expand business processes, invest in better equipment, and improve productivity. However, one caveat is that access to finance alone is insufficient, as it often fails to address other constraints that women face like market access and business training as shown in the studies. More recent studies such as those of Rahman et al. (2018) and Nisa et al. (2020) underscore the importance of complementing financial assistance with capacity-building initiatives to ensure sustainable business growth. Furthermore, sector-specific financial needs vary significantly. For instance, tailoring businesses require investments in sewing machines, quality fabrics, and tools while embroidery enterprises need specialized machines and high-quality threads. On the other hand, basket -weaving operations rely on durable materials such as reeds or bamboo (Islam and Jahan, 2019). Tackling these sectoral differences is therefore essential for effectively supporting rural women’s microenterprises.

Technical training programs are also crucial for enhancing business productivity and driving innovation, particularly in craft sectors and specialized production areas. A study by Hossain et al. (2021) revealed that skills development schemes in rural Bangladesh enhanced product quality and long-term enterprise sustainability. Likewise, Abebe and Schaefer (2019) highlighted how operational efficiency in labor-intensive industries such as woodworking and metalcraft developed as a result of technical training. Nevertheless, the effects of technical support vary by sector and are shaped by gender-specific barriers. Sectors requiring intricate craftsmanship like tailoring and embroidery, for instance, require continuous skills enhancement to maintain design quality and meet consumer demands, while basket weaving relies on advanced techniques to improve durability and aesthetics. On the other hand, food processing enterprises benefit more from advancements in hygiene and packaging techniques. In spite of these requirements, women entrepreneurs experience systemic barriers which include cultural norms that restrict mobility and participation (World Bank, 2020) as well as perceptions regarding the unsuitability of technical development trainings for women (National Training Institute, 2023). In addition, technical training programs often disregard gender-specific needs, such as accommodating women’s time constraints caused by household responsibilities. Thus, these barriers create a gender gap in technical expertise, limiting women’s ability to adopt advanced production techniques, innovate, and compete in broader markets. For these reasons, focusing on the inequalities through inclusive, accessible, and sector-specific training is crucial for advancing gender equity and empowering women entrepreneurs (Chowdhury and Akter, 2019).

In addition to technical programs, management support, and business planning which comprise financial management, strategic planning, and operational efficiency are central to the success of women entrepreneurs. According to Bruhn et al. (2018), enhanced managerial skills lead to improved decision-making and profitability. Similarly, McKenzie and Woodruff (2020) found that structured management training improves business outcomes, particularly for women establishing their enterprises. Research, however, suggests that women often face unique challenges in acquiring and applying managerial skills. In fact, a meta-analysis by Ma et al. (2021) revealed that female entrepreneurs with prior business experience are more likely to engage in formal business planning than their male counterparts, drawing attention to the importance of targeted management training for women. Perceived gender discrimination also affects women’s entrepreneurial activities but as noted by Welsh et al. (2017), improving managerial competencies can mitigate some of the negative effects of discrimination (Pereira et al., 2023). Regardless, sector-specific management needs differ significantly. Tailoring and embroidery businesses, for instance, require advanced inventory and supply chain management to maintain product quality, while basket-weaving enterprises benefit from streamlined production planning to meet seasonal demands. Therefore, tailored management support programs that deal with these sectoral and gender-specific challenges are vital for promoting sustainable business growth.

Marketing support, which encompasses trade shows, digital marketing, and networking, is another essential form of assistance for business growth. Research indicates that accessing broader markets through trade exhibitions and online platforms can significantly increase sales and brand recognition (Bairagi and Ferdousi, 2020; Anwar et al., 2022). However, the effectiveness of these strategies varies in rural women’s micro-enterprises, such as tailoring, embroidery, and basket weaving sectors. While tailoring businesses flourish at local trade fairs where they develop customer relationships and encourage repeat transactions, embroidery enterprises benefit from digital marketing that showcases their unique designs and allows them to access niche markets (Anwar et al., 2022). In contrast, basket-weaving businesses connect with eco-conscious consumers through both trade fairs and digital platforms (Ahmed and Kabir, 2019). Despite these opportunities, rural women entrepreneurs continue to face considerable barriers to marketing, including limited access to networks, inadequate technical skills, and financial constraints (Hasan and Parveen, 2021). Addressing these impediments through targeted marketing strategies is essential for empowering rural women entrepreneurs and furthering sustainable growth.

In summary, the affirmative outcomes of support services for women’s micro-enterprise development rely on addressing sector-specific and gendered needs. As tailoring, embroidery, and basket weaving sectors each require distinct financial, technical, managerial, and marketing support, tailored interventions that consider these unique challenges are crucial for empowering rural women entrepreneurs and ensuring the long-term sustainability of their businesses. Rogers and Gard (2019) and Tekola and Gidey (2019) also emphasized that sector-specific strategies are important for the success of support programs. Generic interventions, however, often fail to deal with the diverse realities of different industries thereby limiting their effects. Therefore, acknowledging sectoral differences is necessary to design targeted support systems that further meaningful growth for women-led micro-enterprises.

### 2.3 Theoretical framework

Building on the wide range of challenges faced by women entrepreneurs, various development interventions such as training, financial services, and marketing programs have been implemented to promote women’s entrepreneurship (Hossain et al., 2021; Yeasmin and Yasmin, 2020). However, the effectiveness of these programs in improving business performance varies considerably. To better understand these variations, Kabeer’s (1999) empowerment framework is useful. The framework conceptualizes empowerment as the interplay among three core components: resources, agency, and achievements. In the context of women’s entrepreneurship, resources include external support women entrepreneurs receive, such as microcredit, training, and marketing assistance, as well as their own abilities, including education and savings that they possess. As discussed above, women tend to have fewer resources than men (Hossain, 2021). The agency component explains how these resources are utilized to lead to achievements. As seen above, often women are not able to utilize the resources effectively, that is, gender norms can restrict women’s mobility and negotiations that they can exercise in their business and how much support they can mobilize from their family and community. Achievements represent the outcomes of these processes, including business performance, income growth, and enterprise sustainability (Bağiş et al., 2023).

Numerous studies have applied Kabeer’s (1999) empowerment framework to examine how support services can be translated to women’s entrepreneurial outcomes, consistently emphasizing that access to resources alone is insufficient. For example, Mahmud et al. (2012) found that microcredit improved women’s business outcomes in rural Bangladesh only when accompanied by decision-making power and family support. Similarly, Ruel et al. (2021) demonstrated that the FAARM program’s impact on empowerment was contingent upon its capacity-building components, which over time enhanced women’s agency. In India, Deshpande and Sethi (2020) found that despite receiving training and credit through government-sponsored programs, many women entrepreneurs struggled to achieve sustained outcomes due to prevailing patriarchal norms that limited their decision-making power and mobility. These studies demonstrate that agency is the critical link between resource access and the achievement of entrepreneurial success.

### 2.4 Research gap

Existing research on micro-enterprises often generalizes findings across different sectors, while failing to account for the distinct challenges faced by women entrepreneurs in specific sectors. Although financial access, technical assistance, and skill development programs are generally referred to in the literature, a focus on evaluating their effectiveness across different micro-enterprise sectors has been inadequate. In general, existing studies have been consistent in examining the overall impact of financial and training programs, but do not take into account whether these interventions work equally well across different types of businesses. For instance, microcredit may provide growth opportunities in one sector while proving insufficient in another if not complemented with relevant training or market access support. Similarly, while marketing support is recognized as beneficial, its success can vary significantly depending on whether a business is product-based or service-based. Given the dearth of sector-specific evaluation, it remains understudied which types of interventions yield the most substantial impact on women’s enterprise income and growth.

This article conducts sector-specific analysis to identify differences in how women micro-entrepreneurs in different sectors translate various resources (training in our study) into achievements (business growth and income in our study).

### 2.5 Support services for women’s micro-enterprise development in Bangladesh

In Bangladesh, many women engage in micro-enterprises as a pathway to overcome structural inequalities in the formal labor market as these ventures provide the flexibility to balance their economic and household responsibilities. Recent trends on women-owned businesses in Bangladesh revealed a steady growth over the years. According to International Finance Corporation (2021), the proportion of women-owned enterprises increased from 7.2% in 2016 to 10% by 2021, indicating progress toward gender inclusivity in entrepreneurship. Moreover, almost all (99.9%) of businesses in Bangladesh are classified as micro, small, and medium enterprises (MSMEs), which contributes approximately 25% to the country’s GDP (Kumar and Suppiah, 2023). These businesses span diverse sectors, comprising agriculture, livestock, fisheries, nurseries, handicrafts, and various non-farm activities. While micro-enterprises contribute significantly to women’s financial independence and household decision-making, the challenges they experience persist such as restricted access to finance, weak market linkages, and insufficient technical knowledge, constraining their long-term sustainability and growth (Hossain and Ahmed, 2020; Ribeiro et al., 2022).

To deal with these barriers, the government and non-governmental organizations (NGOs) have initiated various targeted support services. Major initiatives by the Bangladesh Small and Cottage Industries Corporation (BSCIC) and the SME Foundation provide financial assistance, technical training, and business development services tailored for women entrepreneurs. Other key programs like the SME Foundation’s Women Entrepreneurship Development Program grant subsidized loans and capacity-building training, benefiting thousands of women annually (SME Foundation, 2023; Ahmed et al., 2021b). In addition, microfinance institutions, including Grameen Bank, BRAC, and ASA, collectively disburse millions of dollars in loans each year. BRAC alone assists over 1.3 million women entrepreneurs through its microfinance initiatives, facilitating access to capital for small business ventures (BRAC, 2022). However, while financial access has enabled many women to start businesses, several research studies reveal that access to credit by itself is insufficient without complementary skills development and market exposure (Rahman et al., 2021; Kumar and Suppiah, 2023; Mohsin and Lei, 2020; Ribeiro et al., 2022).

Skills development is another critical component of women’s entrepreneurial success. Thus, the National Skills Development Authority (NSDA) and the Department of Women’s Affairs conduct regular training programs on financial management, digital marketing, and product diversification. Sector-specific training is also provided, such as weaving and embroidery by the Bangladesh Handloom Board, and handicraft skill development by organizations like Tarango and Aarong. These training programs substantially improve business productivity and product quality, particularly in production-intensive enterprises as research indicates (Hossain et al., 2021; McKenzie and Woodruff, 2020; Meyer and Synodinos, 2019). However, disparities in access to training especially for rural women is still a major challenge due to mobility constraints, limited financial resources, and lack of awareness to participate in these initiatives.

Marketing support services are equally important in ensuring the success of women-led micro-enterprises. Participation of women entrepreneurs in trade fairs are facilitated by organizations such as the Export Promotion Bureau (EPB) and SME Foundation providing platforms for them to showcase their products at major events like the National SME Fair and the Dhaka International Trade Fair (Export Promotion Bureau, 2023). In addition, digital marketing initiatives are expanding, with e-commerce platforms integrating rural entrepreneurs into online marketplaces. In spite of these, challenges such as high participation costs, limited digital literacy, and inadequate infrastructure continue to hinder the full integration of women-led businesses into competitive markets (Anwar et al., 2022; Bairagi and Ferdousi, 2020; Rakićević et al., 2016).

Regardless of these efforts, the success of support services diverges across sectors and regions. Microcredit programs, while beneficial, have been criticized for imposing repayment pressures without ensuring business sustainability, particularly for women in lower-income groups (Nisa et al., 2020; Rahman et al., 2018). Similarly, despite advancement in productivity as a result of skills development initiatives, their effects depend on the business. For instance, urban entrepreneurs in service-oriented businesses benefited highly from management training, whereas the effects of technical skills training on rural women involved in handicrafts and textiles have been substantial (Hossain et al., 2021; Ahmed et al., 2021a). Nevertheless, marketing support remains underutilized due to barriers such as lack of capital, digital illiteracy, and limited access to information.

## 3 Materials and methods

### 3.1 Study area

The study focuses on three sectors, tailoring, embroidery, and basket-weaving micro-enterprises, located in Alokdia and Arunkhola Unions in Madhupur Sub-District in Tangail District, Bangladesh. These sectors were selected because they play a significant role in the local economy. Furthermore, they are labor-intensive, accessible to women with limited resources, and reflect the diverse economic activities beyond agricultural work in the study areas. Analyzing these micro-enterprise sectors enhances our understanding of the unique challenges and support needs of women entrepreneurs in rural Bangladesh.

Alokdia and Arunkhola Unions were chosen due to their strategic locations and vibrant markets, where women participated actively in businesses and entrepreneurship. While people in Tangail are primarily reliant on on-farm activities for their livelihood, a significant proportion of women and men are also involved in off-farm activities. Tangail is renowned for its rich handicraft traditions; most notably, the production of Tangail sarees, traditional handloom textiles popular in both domestic and international markets. Furthermore, the district is a hub for micro-scale weaving businesses, where local artisans craft high-quality textiles using hereditary techniques. In addition, tailoring, basket-weaving, and embroidery are key off-farm activities that contribute to the area’s diverse economic fabric. Many of these micro-enterprises are family-run and represent an important part of the local economy.

Although governmental and non-governmental organizations extend support to women in Tangail, they generally experience greater challenges than men in operating their businesses. Moreover, they frequently encounter gender bias within their socioeconomic environment (Ferdousi and Mahmud, 2019).

### 3.2 Selection of organizations

Based on preliminary key informant interviews (KIIs) with Sub-District (upazila) Executive Officer, Sub-District Statistical Officer, and Sub-District Chairperson, this study selected two governmental organizations: the Department of Women’s Affairs (under the Ministry of Women and Children affairs) and the Department of Cooperatives (under the Ministry of Local Government, Rural Development and Cooperatives). Two NGOs: World Vision and Caritas were also chosen since they were actively involved in providing support services for micro-enterprise development in the study areas.

During the scoping field visit, it was observed that these four organizations were actively organizing various programs to promote micro-enterprises. These initiatives included income-generating training, microcredit programs, and awareness-building sessions designed to enhance micro-enterprise skills and sustainability. The Madhupur Sub-District has government-registered organizations administered by both the Department of Women’s Affairs and the Department of Cooperatives. According to the Department of Women’s Affairs Officer, a total of 20 women’s organizations has been registered, each comprising 30 to 35 members actively engaged in micro-enterprises. The Sub-District Cooperative Officer also reported that they have 12 women’s organizations, with each union hosting two registered cooperative organizations, each consisting of at least 60 members. Both unions have four women’s organizations registered under the Department of Women’s Affairs and two cooperative organizations under the Department of Cooperatives. These organizations play a vital role in promoting entrepreneurship and improving the socioeconomic status of rural communities.

The Women’s Affairs Officer and the Cooperative Officer stated that training programs and support services are provided to women entrepreneurs, which are organized directly from their respective offices. The training programs are organized for a duration of 3 months according to the Women’s Affairs Officer, while the Cooperative Officer stated that the training they offer lasts between 1 and 2 months. In addition, the two NGOs, Caritas and World Vision, played a pivotal role in providing entrepreneurship training and microcredit initiatives. Unlike the government organizations, these two NGOs operated through partnerships with registered women’s organizations rather than establishing their own entities. They formed smaller groups of 25–30 members each for weekly training sessions conducted within the union, ensuring accessibility and practical learning opportunities for women entrepreneurs (Table 1).

**Table 1:** Women’s organizations in selected unions of Madhupur Sub-District based on support services rendered by specific government offices and NGOs.

Supporting agency	Arunkhola Union	Alokdia Union
Department of Women’s Affairs	Jolchotro Women’s Organization Ashik Mishik Women’s Organization Pahariful Women’s Organization Aangaria Women’s Organization	Gangail Women’s Organization Maizbari Women’s Organization Dama Basuri Women’s Organization Kathalbari Women’s Organization
Department of Cooperatives	Jolchotro Horisova Nitattik Cooperatives Women’s Organization Gachabari Cooperatives Women’s Organization	Chiring Jolchotro Cooperatives Women’s Organization Shathibari Cooperatives Women’s Organization
Caritas NGO	Caritas Women’s Group	Caritas Women’s Group
World Vision NGO	Women’s Group Organization	Women’s Group Organization

Source: Authors’ field survey in January to June 2017.

In this study, seven types of training (T1–T7) and three types of support services (S1–S3) relevant to the development of women’s micro-enterprises are selected. The seven training programs consist of management-level training, managerial development, market orientation, handicraft skill development, technical knowledge, communication skills, and negotiation training. In addition, the three key support services comprise microcredit, marketing assistance, and production support service. All the programs and services have been crucial in enhancing business sustainability and growth.

The common themes of the training programs for tailoring, embroidery, and basket-weaving sectors in the study areas are typically categorized as follows:

T1. Management development: This type of training emphasizes decision-making ability and confidence in business, financial management, and time management (Flores et al., 2022). In this study, it was found that Department of Cooperatives organized workshops on creating and managing business plans designed for their specific enterprise sector, which assist in the process of designing, launching, and managing a new business. (Brijlal et al., 2014).

T2. Market-Oriented training: This training is regarded as a business philosophy that centers on identifying and meeting customer needs (Meyer and Landsberg, 2021). This study revealed that the Department of Cooperatives arranged training on identifying local and regional markets, networking, and participating in fairs for trading goods and services to meet customer needs.

T3. Handicraft skills development training: For this type of training, the Department of Women’s Affairs has arranged handicrafts skill development training that focuses on enhancing skills in tailoring, embroidery, and basket weaving. It was also observed that women engaged in tailoring received training in modern cutting and stitching techniques, integrating contemporary fashion trends to meet market demands. Apart from this, entrepreneurs in the embroidery sector were trained in intricate stitching styles such as Kantha and Nakshi *Kantha*, which are celebrated for their cultural heritage and commercial appeal. Similarly, women in the basket-weaving sector learned to utilize eco-friendly materials such as jute and banana leaves and craft sustainable and marketable products that aligned with global sustainability trends. These sector-specific training courses not only preserve traditional artistry but also modernize it in accordance with market needs, thereby strengthening the cultural significance and economic viability of handicraft enterprises.

T4. Technical knowledge training: To enhance productivity and product quality, technical knowledge training is conducted using modern tools to upgrade the market segment. Moreover, appropriate teaching methods are employed to ensure higher standards of finished products for competitive markets. By advancing their technical expertise, entrepreneurs can adopt innovative approaches to upgrade their products and increase profitability (Khan and Yasmin, 2020). NGOs World Vision and Caritas arranged technical skills training specific to sectors like tailoring techniques, embroidery patterns, and basket-weaving methods. These sessions generally include modern tools and design trends to enhance productivity and product quality.

T5. Managerial development training: Some training sessions aim to advance managerial abilities by focusing on skills such as planning, communication, time management, and motivation techniques. The study also revealed that the programs organized by the Women’s Affairs government organizations prioritize skills development and personal growth, advancing the participants’ enterprise management skills and their ability to efficiently run their businesses, leading to improved outcomes.

T6. Financial management skills training: This training is central to the development and sustainability of both newly established and existing small businesses (Kirsten, 2018). The research also found that women entrepreneurs across all three sectors tailoring, embroidery, and basket-weaving received some guidance on fundamental financial practices. The Cooperative government organization establishes training that prepares them in financial planning, bookkeeping, and in handling financial tools effectively. Thus, training is designed to facilitate their advancement in managing their micro-businesses.

T7: Communication and Negotiation: To provide effective communication and negotiation training, organizations customize programs to address the unique challenges that confront women entrepreneurs in stakeholder interactions and build their confidence (Khalique et al., 2021). The study found that two NGOs: World Vision and Caritas organized expert trainers to conduct interactive workshops, complemented by virtual sessions and video tutorials for improved accessibility.

S1. Production support services: In a year, one-time cash donation grants and essential production support services such as production-related materials (e.g., sewing machine) are also provided by the Department of Women’s Affairs and the Cooperative government organizations to entrepreneurs. In addition, workshops were conducted to improve production processes, manage resources efficiently, and expand production efficiency and product quality. Khan and Rahman (2018) indicated that these resources can lead to a reduction in production costs, improved scalability, and empower entrepreneurs to meet market demands effectively.

S2. Marketing support services: This type of support services include trade fairs organized by both the Women’s Affair and the Cooperative government organizations in local areas to assist entrepreneurs in showcasing and marketing their products. As part of their services, entrepreneurs are provided with shop allocations and financial assistance to further their market exposure and sales opportunities.

S3. Micro-credit support services: Two distinct types of micro-credit services are long-term, which extend over a span of 2 years, and short-term, which is limited to 1 year. Each of the government organizations and NGOs apply its own unique criteria and conditions in extending these credits.

### 3.3 Data collection and sampling

This study employs a multi-method research (MMR) design, integrating quantitative and qualitative approaches within a single paradigm. Quantitative data were collected through a structured survey with women entrepreneurs, while qualitative insights were obtained from in-depth and key informant interviews. This integrated approach allowed for a comprehensive analysis of the factors influencing women-led micro-enterprises in rural Bangladesh.

The survey population was those who had received training and support services from government offices and NGOs. According to data from the Sub-District Statistical Office of Madhupur, approximately 4,000 male and female entrepreneurs received support from governmental and non-governmental organizations during the 3 years preceding this study (2014–2017). Of these, 2,600 were women entrepreneurs engaged in home-based micro-enterprises, primarily in the Alokdia and Arankhola unions. Specifically, 1,450 women (36.3%) operated in Arankhola, and 1,150 (28.8%) in Alokdia (Sub-district Statistical Office, 2017). These two unions were selected as the study sites due to their high concentration of women-led micro-enterprises and substantial access to support services, including training, microfinance, raw materials, and market linkages, which play a critical role in sustaining and scaling their businesses.

Following Yamane’s formula (Yamane, 1976), a minimum suggested sample size of 170 was determined using a 10% margin of errors. A stratified random sampling method was employed to select respondents, with the sample size proportionally allocated based on the population of female entrepreneurs in each union. Accordingly, 95 respondents were selected from Arankhola and 75 from Alokdia.

This sampling framework ensures that the findings reflect the actual distribution of supported women entrepreneurs across the two unions and the three key sectors.

To complement the quantitative findings, the study included 20 in-depth interviews (IDIs) with women entrepreneurs to capture a range of lived experiences and challenges. These participants were selected from among the 170 survey respondents in the study areas, based on their receipt of awards between 2014 and 2017, including the Best Joyita Award, SME Fair Prize, NGO Fair Prize, Development and Business Fair Prize, and the BSCIC Boishakhi Fair Prize. Of the 20 participants, eight were from Arankhola Union and 12 from Alokdia Union. All names used in this study are pseudonyms. In addition, nine key informant interviews (KIIs) were conducted with the Sub-District Executive Officer, Women Affairs and Cooperative officers, two NGO representatives, the Sub-District Chairperson, Vice-Chairperson, and two Union Chairpersons. These interviews provided institutional perspectives on program implementation, effectiveness, and gaps in service delivery.

All in-depth interviews (IDIs) and key informant interviews (KIIs) participants were anonymized. Pseudonyms were used instead of real names, and participant details (such as age or occupation) were included only when relevant and non-identifiable. Responses were coded by sector and role (e.g., embroidery, tailoring, and basket-weaving trainees) to preserve the context while maintaining ethical standards. All qualitative data were stored securely and only accessed by the researcher.

### 3.4 Data analysis

In 2017, data on business income for 2014 prior to receiving support services were collected based on the recall method. Similar types of data for 2017 after obtaining the services were also gathered. The following conversion was applied to calculate the real income for 2017 in order to compare it with the 2014 income (Mendez-Carbajo, 2023).


$$\begin{array}{l} \text{Real Income}\;(2017)=\text{Nominal Income}\;(2017)\times \\ \text{Conversion Factor}\;(\mathrm{CF}) \end{array}$$

where


$$\mathrm{CF}=\frac{\mathrm{CPI}(2014)}{\mathrm{CPI}(2017)}$$

and the Consumer Price Index (CPI) of Bangladesh was 136.13 at the end of 2014 and 152.53 at the end of 2017 (Base Year 2010)^1^.

Income normalization is employed to emphasize growth in productivity rather than size (Subramanyam, 2014). This allows for a comparison of financial performance among enterprises on a common scale, independent of their size. To calculate normalized income for 2014 and normalized real income for 2017, income is divided by enterprise size (the number of employees) as follows:


$$\text{Normalized}\;\;\text{Income}\;(2014)=\frac{\;\text{Income}\;(2014)}{\text{Enterprise Size}\;}$$

$$\text{Normalized real Income}\;(2017)=\frac{\text{Real Income}\;(2017)}{\text{Enterprise Size}\;}$$

The increase in annual normalized real income is used as the dependent variable, which is generated by subtracting the 2014 normalized income from the 2017 normalized real income .

Frequency distribution is used to illustrate the reasons behind women’s involvement in micro-enterprise businesses. The socioeconomic characteristics of women entrepreneurs (e.g., increased i**ncome,** age **group** and marital status) are presented using cross-tabulation. Paired *t*-test was performed to examine the statistical significance of the growth in mean normalized real income from 2014 to 2017. To examine the correlations, the point bi-serial correlation (Gupta, **1960**) was determined between the increase in normalized income and dichotomous variables such as training, support services, and other covariates. Likewise, Pearson correlation was applied to numerical variables like years of experience in business while **Spearman rank correlation** was employed on ordinal **variables such as education**al **level** and **age group.** The Variance Inflation Factor (VIF) was calculated to assess potential multi-collinearity whereby a VIF value exceeding 10 typically indicates problematic multi-collinearity (O’Brien, 2007).

Finally, ordinary least squares (OLS) regression was performed to identify the effects of organizational training and support services on the growth in normalized real income while controlling for covariates. The variables included in this analysis are presented in Table 2.

**Table 2:** Variables included in the ordinary least squares regression analysis.

Definition	Variable	Measurement/Unit
Dependent variable		
Normalized Real Income in 2017- Normalized Income in 2014	Increase in income	Bangladesh Taka (BDT)
Business variables		
Previous business experience	Business background of family member	1 if Yes, 0 if No
Years of business experience	Year of experience in business	Years
Receiving family Support	Family support for doing business	1 if Yes, 0 if No
Investing time in business	Investment of time in your business	Years
Access to long-term microcredit (2 years)	Access to long term (2 years) micro-credit	1 if Yes, 0 if No
Access to short-term microcredit (1 year)	Access to short term (1 year) micro-credit	1 if Yes, 0 if No
Received organizational training		
T1. Management development training	Management development training	1 if Yes, 0 if No
T2. Market-Oriented training	Market-Oriented Training	1 if Yes, 0 if No
T3. Handicraft skills development training	Handicraft skills development training	1 if Yes, 0 if No
T4. Technical knowledge training	Technical knowledge training	1 if Yes, 0 if No
T5. Managerial skills development training	Managerial skills development training	1 if Yes, 0 if No
T6. Financial management skills training	Financial management skills training	1 if Yes, 0 if No
T7. Communication skills development training	Communication skills development training	1 if Yes, 0 if No
Receiving support services		
S1. Production related material	Production related material	1 if Yes, 0 if No
S2. Financial support to attend trade fair	Financial support for attending local trade fair	1 if Yes, 0 if No
S3. Stall allotment at local trade fair	Stall allotment to attend trade fair	1 if Yes, 0 if No
Covariates		
Marital Status of the respondent	Marital Status	0 if single (unmarried, divorced, widow), 1 if married
Age of the respondent	Age	Ordinal (dummy coded): Young (18–30), Middle (31–45), Old (46–60)
Sector of the business	Sector	Categorical (dummy coded): Tailoring, Embroidery, Basket weaving
Education level of the respondent	Education	Ordinal (dummy coded): Primary (below 5th) Secondary (5th to 10th), High School and Above (10th+)

Source: Authors’ field survey in January to June 2017.

T**o include categorical variable**s **in** the analysis, nine dummy **(**dichotomous**)** variable**s** were defined for three ordinal variables **(**entrepreneurial occupation, **age group and** e**ducation level)** in the following manner.


$$D_{k}=\{\begin{array}{c} 1:\text{if the respondent belongs to that}\\ \text{particular category or level}\\ 0:\text{otherwise} \end{array}$$

where $D_{k}$ indicates the dummy variable representing one level or category. The base category/level was “young” for age group and “primary” for education level (Yip and Tsang, 2007).

The OLS regression model is expressed as follows:


$$\begin{array}{l} Y_{j}=a+\sum b_{i}*T_{\mathrm{ij}}+\sum c_{k}*D_{\mathrm{kj}}+\sum v_{\mathrm{ik}}*T_{\mathrm{ij}}*D_{\mathrm{kj}}+\\ \sum g_{\mathrm{im}}*T_{\mathrm{ij}}*S_{\mathrm{jm}}+\sum z_{p}*E_{\mathrm{pj}}+u_{j} \end{array}$$

where $Y_{j}$ represents the increase in normalized real income of the *j*-th respondent. The term a denotes the *Y*-intercept; $b_{i}$ represents the coefficient of the *i*-th training and support service dummy; $T_{\mathrm{ij}}$ is a dummy variable that indicates whether the *j*-th respondent received the *i*-th type of training or support service; $c_{k}$ refers to the coefficient of the *k*-th business sector; $D_{\mathrm{kj}}$ is a dummy variable representing the *k*-th business sector; $v_{\mathrm{ik}}$ denotes the interaction coefficient of the *i*-th training dummy and *k*-th business dummy; $g_{\mathrm{im}}$ is the interaction coefficient of the *i*-th training dummy and the *m*-th level of education; $S_{\mathrm{jm}}$ is the dummy representing the education level *m*; $z_{p}$ is the coefficient of the *p*-th covariate; $E_{\mathrm{pj}}$ denotes the *p*-th covariate; and $u_{j}$ is the random error term.

The effects of interacted variables are evaluated by jointly using the coefficients of both the original variables and the interaction terms. For instance, for entrepreneurs in the primary education level, the training effect is calculated as the sum of the direct coefficient of the training variable (i.e., $b_{i}$ ) and the interaction coefficient between the training dummy and the relevant business sector dummy (i.e., $v_{\mathrm{ik}}$ ). In other words, the effect of training *i* in sector *k* is given by $b_{i}+v_{\mathrm{ik}}$ . For example, the effect of management-level training in primary-level educated tailoring entrepreneurs is the sum of the coefficient of management-level training and the interaction coefficient between the management-level training dummy and the tailoring business dummy. For entrepreneurs in a higher education level, the effect is calculated by further adding the interaction term between the training dummy and the relevant education level dummy (i.e., $g_{\mathrm{im}}$ ), rendering $b_{i}+v_{\mathrm{ik}}+g_{\mathrm{im}}$ . For instance, the effect of management-level training in higher-level educated tailoring entrepreneurs is given by the sum of the three coefficients: management-level training, the interaction between management-level training and the tailoring sector, and the interaction between the management-level training and higher education.

In the calculation, the statistically insignificant coefficients are regarded as being not different from zero. Given the modest sample size and the nature of the data collection method, the *p*-value threshold of 0.10 was adopted for statistical significance.

## 4 Results and discussion

### 4.1 Profile of the respondents

Identifying the demographic profile of women entrepreneurs is essential to contextualize the findings of this study. Table 3 presents the key characteristics such as age, educational level, and marital status across the three micro-enterprise sectors, tailoring, embroidery, and basket-weaving. These factors influence business performance, access to financial resources, and the participation of respondents in training programs, thereby shaping overall income growth trends.

**Table 3:** Demographic characteristics of women entrepreneurs by sector (*n* = 170).

Characteristic	Category	Tailoring (*n* = 63)		Embroidery (*n* = 73)		Basket weaving (*n* = 34)	
		*n*	%	*n*	%	*n*	%
Age group	Young (18–30)	32	50.8	24	32.9	12	35.3
	Middle (31–45)	23	36.5	35	47.9	12	35.3
	Older (46–60)	8	12.7	14	19.2	10	29.4
Educational attainment	Primary School or below	18	28.6	32	43.8	22	64.7
	Secondary School	15	23.8	19	26.0	4	11.8
	High School or above	30	47.6	22	30.1	8	23.5
Marital status	Single	25	39.7	33	45.2	8	23.5
	Married	38	60.3	40	54.8	26	76.5

Source: Authors’ field survey in January to June 2017.

### 4.2 Types of support services and training received by the respondents

Women entrepreneurs in the Madhupur Sub-district of Tangail District in Bangladesh received training and support services (SS) from 2014 to 2017 that were designed to develop their micro-enterprises. These initiatives offered vital resources and skill-building opportunities, empowering the women to enhance their entrepreneurial skills and contribute to local economic growth.

Table 4 presents the various organizational training and support services received by women entrepreneurs, highlighting the availability of diverse training options for micro-enterprises in the tailoring, embroidery, and basket weaving sectors. The adoption of organizational training reflects the unique needs of each sector.

**Table 4:** Respondents in each sector by support services (%) (*n* = 170).

SS type	SS sub-type	Tailoring (*n* = 63)	Embroidery (*n* = 73)	Basket weaving (*n* = 34)
Micro-enterprise development training	T1. Management development Training	57.1	58.9	41.2
	T2. Market-Oriented Training	60.3	53.4	44.1
	T3. Handicraft Skill Development Training	47.6	41.1	44.1
	T4. Technical Knowledge Training	58.7	46.6	29.4
	T5. Managerial skill Development Training	41.3	50.0	41.2
	T6. Financial Management skills Training	28.6	16.7	20.6
	T7. Communication and Negotiation	39.7	55.6	52.9
Production support service	S1. Production Related Materials	82.5	65.8	70.6
	S2. Donation	12.7	2.7	0.0
	Not Applicable	17.5	34.2	29.4
Marketing support services	S3. Financial Support to Participate in trade fair	47.6	45.2	38.2
	S4. Allotted Stall to Participate in trade fair	34.9	42.5	44.1
	Not Applicable	25.4	32.9	29.4
Access to micro-credit	S5. Long-term (2 years) micro-credit	63.9	69.5	59.3
	S6. Short-term (1 year) micro-credit	29.5	23.3	37.0

Source: Authors’ field survey in January to June 2017.

The management development training received from the Department of Cooperatives was found to be highly utilized in embroidery (59%), followed by tailoring (57%) and basket weaving (41%). The market-oriented training followed a similar pattern, with 60% in tailoring, 53% in embroidery, and 44% in basket weaving. This indicates the importance of management-development and marketing skills training arranged by the government’s cooperative organizations in the sectors.

For the handicraft skills development training, approximately 48% of the respondents in tailoring received the training from the Department of Women’s Affairs. This was followed by basket-weaving and embroidery sectors at 44 and 41%, respectively. Technical knowledge training provided by NGO World Vision and Caritas was highly received by those in tailoring (59%) and embroidery (47%). About 29% of the respondents in the basket-weaving sector joined the training, which was the lowest proportion.

The managerial development training organized by Department of Women’s Affairs was attended by 50% of the respondents in the embroidery sector while 41% of the women in tailoring and basket weaving participated in the training. As for the communication and negotiation training, a significant proportion of the women entrepreneurs in the embroidery attended (56%) while those in basket weaving sector received the training (53%) which was arranged by NGO World Vision. In contrast, the tailoring businesses (40%) received communication and negotiation training from NGO Caritas. Comparatively few women entrepreneurs participated in the financial management training arranged by Department of Cooperatives government organization; 29% in tailoring, 17% in embroidery, and 21% in basket-weaving.

A significant majority of respondents received production support services. Most of the respondents in the tailoring business (83%) received materials, such as sewing machines, while only 13% received cash donation from the Department of Women’s Affairs to support their business. Around 18% of the respondents did not receive any production support services. As for those in the embroidery sector, around 66% received production support services, while just 3% received cash donations from the Department of Women’s Affairs. Women entrepreneurs who did not receive any type of production support services account for 34% of the total respondents. In the basket weaving business, a very high proportion of respondents (71%) received production support services from the Department of Cooperatives, while the rest (29%) did not receive production support services.

Regarding marketing support services including financial assistance and allotted stalls, the study identified 48% in the tailoring sector who confirmed receipt of financial support from the Department of Women’s Affairs. Likewise, around 35% of the respondents in the sector benefited from the provision of allotted stalls delivered by the organization. On the other hand, 25% of the tailoring entrepreneurs received no marketing support. As for those in the embroidery sector, some respondents (45%) received financial assistance, while 43% were allocated stalls at trade fairs by the Department of Women’s Affairs. On the contrary, about one-third of the entrepreneurs reported not receiving any support. Entrepreneurs in the basket weaving sector reported being granted some financial support (38%) and 44% benefited from stalls which were provided by the Department of Cooperatives while 29% received no marketing support. These statistics indicate the varying levels of support received across the three sectors.

Findings from the study also revealed that the Department of Women’s Affairs offered microcredit support services to the entrepreneurs. In particular, around 64% of the respondents in the tailoring sector obtained long-term microcredit from the organization, while about a third (30%) secured short-term microcredit from the NGO Caritas. Similarly, in the embroidery sector, a majority of the entrepreneurs (70%) obtained long-term microcredit from Department of Women’s Affairs, while 23% benefited from short-term loans offered by Caritas. More than half of the entrepreneurs (59%) in the basket-weaving sector acquired long-term microcredit from NGO Caritas, while 37% of them received short-term microcredit from NGO World Vision. In light of these findings, the Sub-District Cooperative Officer stated that:

*Microcredit disbursement depends on business sustainability, profitability, and repayment capacity. Businesses like tailoring and embroidery, which have steady demand and predictable income, are more likely to receive long-term microcredit due to lower financial risks. However, basket-weaving businesses experience seasonal demand fluctuations and longer production cycles, making their income less stable. Due to these uncertainties, we consider them higher-risk borrowers and exercise caution, often granting short-term credit rather than long-term loans (KII (Key Informant Interviews), 2017).*

Table 5 shows that only about 38–41% of respondents across tailoring, embroidery, and basket weaving enterprises reported having a family business background, while 45–53% received active family support for their enterprises. These figures highlight that while family entrepreneurial experience was not widely available, family support in practice played a slightly stronger role, particularly in basket weaving (53%). The IDIs confirm that having a family business background did not automatically translate into direct benefits, while women entrepreneurs have to bear the household burden and restricted mobility. Many women explained that even if their family engaged in business, their experience was not easily transferred to women due to gendered expectations/mobility. Regarding this, Khodeja Khatun, a 34-year-old embroidery entrepreneur (respondent 16) shared:

**Table 5:** Percentage of respondents with family business background and family support.

Variable	Entrepreneurs’ occupation		
	Tailoring	Embroidery	Basket -weaving
Business background of family member (1 if yes, 0 if no)	41.3	41.1	38.2
Family support for business (1 if yes, 0 if no)	47.6	45.2	52.9

Source: Authors’ field survey in January to June 2017.

*“My father had a small shop where he would do embroidery business. But he never taught me about it as I am a woman. As a woman, I was not expected to join him due to restricted mobility as well as household burden. I had to learn by myself when I started my own work.”*

This reflects Kabeer’s (1999, 2005) argument that access to resources (in this case, family knowledge) is mediated by gender norms for women to utilize them.

By contrast, family support for ongoing business activities had a more direct influence. Women repeatedly emphasized in IDIs that without spousal or parental support, it was difficult to manage business commitments alongside household responsibilities. Concerning this, Amena Khatun, a 29-year-old tailoring entrepreneur (respondent 14) shared:

*“My husband helps me by buying cloth from the market and supports my decision which enables me to do my business and apply managerial and market skills effectively. Without his help, I cannot leave the house easily. This support is more important than whether my family had a business before.”*

Similarly, a basket weaver named Shirin Aktar a 42-year-old basket weaver (respondent 15), highlighted how her sons supported her enterprise:

*“They bring bamboo from far away. I cannot carry it. Because they help me, I can continue weaving. However, due to seasonal volatility and social undervaluation, I faced challenges including seasonal demand, poor market recognition, and restricted access to raw materials like bamboo or cane”.*

These accounts demonstrate how family support enhances women’s agency by enabling mobility, access to input, and time allocation factors critical for business performance.

Table 6 presents the descriptive statistics of continuous response variables for entrepreneurs across the three sectors. The average years of business experience were highest among basket-weaving entrepreneurs (Mean = 12.1, Standard Deviation (SD) = 2.3), followed by tailoring (Mean = 8.6, SD = 2.0) and embroidery (Mean = 7.4, SD = 2.8). This indicates that basket weavers, on average, had substantially longer entrepreneurial experience compared to their counterparts in tailoring and embroidery. Regarding time investment, entrepreneurs in embroidery (Mean = 8.4, SD = 1.7) and basket weaving (Mean = 8.5, SD = 1.8) devoted slightly more hours per day to their businesses than those in tailoring (Mean = 7.9, SD = 1.6). These sectoral variations in experience and time commitment may reflect differences in production cycles, labor intensity, and market demand (Ladzani and van Vuuren, 2018; Minniti and Naudé, 2010).

**Table 6:** Business experience and time investment by sector.

Variable	Statistic	Entrepreneurs’ occupation		
		Tailoring	Embroidery	Basket Weaving
Experience in business (Years)	Mean	8.6	7.4	12.1
	SD	2.0	2.8	2.3
Investment of time in your business (in hours)	Mean	7.9	8.4	8.5
	SD	1.6	1.7	1.8

Source: Authors’ field survey in January to June 2017.

### 4.3 Income growth in women-owned micro-enterprises (2014–2017)

The *t-*test analysis presented in Table 7 provides the level of income increase experienced by women entrepreneurs in tailoring, embroidery, and basket weaving as a result of their participation in training programs and support services between 2014 and 2017. A comparative analysis of income growth across sectors reveals distinct patterns. Firstly, tailoring businesses exhibited the highest absolute income growth of BDT 45,417 (26.9%). Embroidery enterprises also attained increase in BDT 42,098, the highest percentage growth at 32.2%, while basket weaving displayed the highest income variability but still posted a significant increase of BDT 39,314 (29.2%)^2^.

**Table 7:** Increase in income of women-owned microenterprises (2014–2017) in rural Bangladesh: the paired *t*-test (*n* = 170).

Types of Business sectors	Source	Mean	Std. Dev.	*t*-statistic
Tailoring (*n* = 63)	2017 normalized real income	214,581	50,918	67.7***
	2014 normalized income	169,163	49,826	
	Change	45,417	5,323	
Embroidery (*n* = 73)	2017 normalized real income	172,843	52,690	43.8***
	2014 normalized income	130,745	45,737	
	Change	42,098	8,213	
Basket weaving (*n* = 34)	2017 normalized real income	173,988	67,060	34.0***
	2014 normalized income	134,674	62,931	
	Change	39,314	6,740	

^***^ Statistically significant at 1% level. Source: Authors’ Field Survey in January to June 2017.

### 4.4 Determinants of income growth among women entrepreneurs by sector

Table 8 indicates that all variables had VIF values below the conservative threshold of VIF < 3 (Hair et al., 2010; Kutner, 2004). This confirms that there was no severe multicollinearity, and all independent variables were thus retained in the model.

**Table 8:** Variance inflation factor (VIF) among the independent variables in the regression analysis (*n* = 170).

Variable	VIF
Business background of family member (1 if Yes, 0 if No)	1.4
Experience in business (Years)	1.7
Family support for business (1 if Yes, 0 if No)	1.2
Investment of time in your business (Hours)	1.3
Marital status (1 if married, 0 Single)	1.2
Middle age (Dummy)	1.4
Older age (Dummy)	1.5
Secondary education (Dummy)	1.7
Higher education (Dummy)	1.4
Tailoring sector (Dummy)	2.5
Embroidery sector (Dummy)	2.1
Utilizing long-term (2 years) micro-credit (Dummy)	1.4
Utilizing short-term (1 year) micro-credit (Dummy)	1.4
Management development training (Dummy)	1.6
Market-oriented training (Dummy)	1.8
Handicraft skill development training (Dummy)	1.5
Technical knowledge training (Dummy)	1.4
Managerial skill development training (Dummy)	2.2
Financial management skills training (Dummy)	1.9
Communication and negotiation training (Dummy)	2.7
Production-related materials (Dummy)	1.6
Financial support to attend local trade fair (Dummy)	1.9
Stall allotment at local trade fair (Dummy)	1.6

Source: Authors’ field survey in January to June 2017.

Table 9 presents the results of the regression analysis. The *R*^2^ is 0.782, indicating that the majority of the variance in income growth is explained by the set of independent variables included in the model. Experience in business has had positive effects on normalized real income. For a one-year increase in experience, income increased by BDT 618 on average, holding other variables unchanged. Family support had positive effects as well, where the presence of family support led to an additional increment by BDT 1,567.

**Table 9:** Determinants of the increment in normalized real income among women entrepreneurs in rural Bangladesh: ordinary least squares (*n* = 170).

Variable	Coeff.	Std. errors	*p*-value
Business background of family member (1 if Yes, 0 if No)	678.6	873.5	0.439
Experience in business (years)	617.9***	203.3	0.003
Family support for business (1 if Yes, 0 if No)	1,566.5^*^	843.1	0.066
Investment of time in your business (In hour per day)	380.5	246.0	0.125
Marital Status (1 if married, 0 otherwise)	698.0	966.9	0.472
Middle age (Dummy)	741.2	1,205.6	0.540
Older age (Dummy)	–1,001.5	1,314.9	0.448
Secondary education (Dummy)	659.1	4,842.8	0.892
Higher education (Dummy)	971.2	5,054.4	0.848
Tailoring (Dummy)	1,434.8^**^	664.0	0.033
Embroidery (Dummy)	2,706.2^*^	1,438.3	0.063
Utilizing long-term (2 years) micro-credit (Dummy)	2,301.7***	836.5	0.007
Utilizing short-term (1 year) micro-credit (Dummy)	165.9	121.5	0.175
Management development training (Dummy)	2,015.7^**^	940.9	0.035
Market-oriented training (Dummy)	1,676.8	2,560.6	0.514
Handicraft skills development training (Dummy)	311.5	187.4	0.100
Technical knowledge training (Dummy)	1,678.1***	588.8	0.005
Managerial skill development training (Dummy)	1,528.9^*^	837.0	0.071
Financial management skills training (Dummy)	1,615.8	1,027.9	0.119
Communication and negotiation training (Dummy)	1,444.3^*^	793.5	0.072
Production-related materials (Dummy)	3,422.7***	1,010.7	0.001
Financial support to attend local trade fair (Dummy)	265.2	275.0	0.337
Stall allotment at local trade fair (Dummy)	1,360.2^**^	553.8	0.016
Secondary education*Management development training	336.3	2,811.0	0.905
Secondary education*Market-oriented training	198.9	2,550.8	0.938
Secondary education*Handicraft skill development training	742.2^*^	413.9	0.076
Secondary education*Technical knowledge training	−812.5^*^	442.0	0.069
Secondary education*Managerial skill development training	1,576.9	3,371.8	0.641
Secondary education*Financial management skills training	3,144.6	4,466.7	0.483
Secondary education*Communication and negotiation training	4,806.5	3,560.8	0.180
Higher education*Management development training	568.9	2,228.6	0.799
Higher education*Market-oriented training	3,214.4^**^	1,327.6	0.017
Higher education*Handicraft skill development training	1,066.1^*^	563.8	0.061
Higher education*Technical knowledge training	−557.8^**^	223.1	0.014
Higher education*Managerial skill development training	−2,035.1	1,823.6	0.267
Higher education*Financial management skills training	−2,049.0	2,794.0	0.465
Higher education*Communication and negotiation training	−801.3	2,638.8	0.762
Tailoring*Management development training	1,260.0^*^	675.1	0.065
Tailoring*Market-oriented training	−1,578.6	3,042.7	0.605
Tailoring*Handicraft skill development training	1,255.3^**^	592.1	0.036
Tailoring*Technical knowledge training	3,529.9	2,892.0	0.225
Tailoring*Managerial skill development training	3,342.8^**^	1,627.6	0.043
Tailoring*Financial management skill training	−1,088.0***	613.1	0.079
Tailoring*Communication and negotiation training	791.8	4,204.8	0.851
Embroidery*Management development training	−785.6	2,543.0	0.758
Embroidery*Market-oriented training	−670.2	2,905.2	0.818
Embroidery*Handicraft skill development training	1,102.8	664.3	0.100
Embroidery*Technical knowledge training	708.8^*^	356.9	0.050
Embroidery*Managerial skill development training	1,421.3	3,602.6	0.694
Embroidery*Financial management skills training	−413.8	323.7	0.204
Embroidery*Communication and negotiation training	537.0	3,639.7	0.883
Constant	13,192.1***	2,284.3	0.000
*R* ^2^	0.782		
*F*-statistics (*p*-value)	8.3		0.000

^***^, ^**^, and ^*^ indicate *p* < 0.01, < 0.05, and < 0.10, respectively. Source: Authors’ field survey in January to June 2017.

Short-term micro credit did not have significant effects, while the use of long-term microcredit increased the normalized real income by BDT 2,302. Farhana Yasmin, a 32-year-old woman in the tailoring sector and the chairperson of the Ashik Mishik Women’s Organization in Arunkhola (Respondent 25), shared her experience:

*“I have been involved in the tailoring business for a long time, but initially, I faced numerous challenges, particularly a lack of capital. After receiving training from the Sub-District Women’s Affairs Office and the Cooperative Office, I applied for and received microcredit (two years duration) from the Women's Affairs Office. This duration of micro-credit support service helped me to purchase materials and start production.”*

Financial support to attend local trade fairs did not make a significant difference in income. In contrast, production-related materials and stall allotment at local trade fairs had positive effects being +BDT 3,423 and +BDT 1,360, respectively. This was reinforced by the qualitative account of Nazma (Respondent 30), a 30-year-old tailoring entrepreneur and recipient of the Joyita Prize:

*“Participating in government-organized trade fairs allowed me to showcase my products to new buyers. It not only helped boost my sales but also gave me the confidence to expand my customer base and adopt better product presentation strategies.”*

Business background of family members, time investment in business, marriage, and age had no significant effect on average. Notably, education alone had no significant effect on income either, though it showed synergy with some of the training programs as explained below.

#### 4.4.1 Tailoring sector

In the tailoring sector, women entrepreneurs benefited from training programs on management (+3,276 = +2,016 + 1,260), handicraft skills development (+1,255 = 0 + 1,255), technical knowledge development (+1,678 = +1,678 + 0), managerial skills development (+4,872 = +1,529 + 3,343), and communication & negotiation (+1,444 = +1,444 + 0), according to the interaction terms. On the other hand, market-oriented training had no significant effect, while financial skills development training had negative effects (−1,088 = 0–1,088), which were observed only in the tailoring sector. This effect sizes indicated in the parentheses apply to those women with education below secondary school level because of the interaction terms with education levels. Among the different types of training, managerial skills development had the largest effect on income for those with education below the secondary level.

This was supported by Asma Aktar, a 28-year-old woman in the tailoring sector (respondent 21), a less-educated tailoring entrepreneur who reported:

*“I cannot read much, but the training on keeping costs and managing customers helped me earn more than before. I lack formal schooling, but support from my family allowed me to apply these new skills in my home-based tailoring work, which increased both my income and confidence.”*

For those women with secondary school education, the effects of handicraft skills training were larger (+1,998 vs. +1,255) compared to those with education below secondary level. This suggests that secondary school education has synergistic effects with handicraft skills training.

Farhana Islam (Respondent Number 12), a 29-year-old tailoring entrepreneur with a Secondary School Certificate and recipient of the Successful Women Entrepreneur Award from Transparency International Bangladesh (2013), shared her experience, highlighting the effects of handicraft skills training:

*"Before the training, I was only able to do basic stitching. But after the handicraft training, I improved a lot in design and finishing. Since I completed school, I can follow the instructions well.”*

Her case reflects how secondary education strengthens the impact of training by improving literacy, comprehension, and problem-solving skills. These capabilities are particularly valuable in tailoring, where entrepreneurs must understand patterns, apply measurements accurately, and incorporate creative designs. Consequently, women with secondary education are more concentrated in tailoring enterprises because this sector demands higher literacy and technical adaptability than embroidery or basket weaving, where basic manual skills are often sufficient.

On the other hand, technical knowledge training was less effective for those with secondary school education (+866 vs. +1,678), possibly because educated women could already grasp and apply much of the technical content without additional support.

Education at high school level or higher in tailoring sector shows the effects with handicraft skills training (+2,321 vs. +1,255), and market-oriented training (+3,214 vs. 0), greater gains from certain training programs compared to those with only secondary or primary education. In contrast this level of education shows the same effect with management-level training (+3,276 vs. +3,276), and communication negotiation training (+1,444 vs. +1,444). However, training with technical knowledge (+1,120 vs. + 1,678) was less effective for those with higher school education.

Fatema Begum, (Respondent Number 2), a 30-year-old tailoring entrepreneur and recipient of the prestigious Joyita Prize in 2015 from the Department of Women Affairs, shared her reflections on the transformative effects of training programs. With a high school education, she reported that the handicraft skills training (+2,321 vs. +1,255), and market-oriented training (+3,214 vs. 0), were particularly beneficial, stating that her educational background enabled her to easily follow the sessions and apply the knowledge to improve her business operations. Fatema shared:

*“The training enhanced my ability to organize my work more efficiently. I could follow the sessions easily and deal with customers more confidently”.*

She also highlighted the relevance of the market-oriented training, noting that while others may have found it less helpful, it resonated with her and complemented the concepts introduced during the broader training program. She emphasized the relevance of the market-oriented training, explaining that while others sometimes found it less beneficial, she was able to connect it with concepts introduced in broader training programs. According to her, her education enabled her to absorb more advanced training content, which in turn led to noticeable improvements in managing and growing her enterprise. She concluded that continued access to such targeted support would further strengthen the business prospects of women entrepreneurs like herself.

#### 4.4.2 Embroidery sector

In the embroidery sector, women entrepreneurs benefited from training programs on management (+2,016 = +2,016 + 0), technical knowledge development (+2,387 = +1,678 + 709), managerial skills development (+1,529 = +1,529 + 0), and communication (+1,444 = +1,444 + 0). Market-oriented training, handicraft skills training, and financial skills training had no significant effect. This effect sizes indicated in the parentheses apply to those women with education below secondary school level.

The results indicate that income growth in the embroidery sector is strongly influenced by technical skills development, followed by management-level training.

This quantitative finding was echoed in the qualitative interviews. Shahana (Respondent 11), a 28-year-old embroiderer with a primary school education, explained:

*“I received technical skill development training from Caritas NGO. Before the training, my designs were very simple, and I could only copy what I had seen from others. During the training, I learned new stitching patterns and how to combine colors in better ways. The NGO also followed up with us afterwards, which helped a lot. Customers noticed the difference in my work and started placing more orders. Now I am able to charge a higher price for my products.”*

Shahana’s account illustrates how technical skill development training directly improved product quality and marketability in the embroidery sector. The follow-up support provided by the NGO further reinforced the application of these skills in practice, ensuring that the training was not merely theoretical but translated into sustained improvements in production. This supports the quantitative findings, which identified technical training as the strongest driver of income growth among embroidery entrepreneurs, with effectiveness shaped not only by education but also by the presence of close follow-up.

With secondary school education in embroidery sector, the effects of handicraft skills training became significant (+742 vs. 0) compared to those with education below secondary level. This suggests that secondary school education has synergistic effects with handicraft skills training. On the other hand, technical knowledge training was less effective (+1,574 vs. +2,387) for those with secondary school education. The effects of other training programs were not moderated by secondary school education.

The quantitative evidence, as far as handicraft training is concerned, is well supported by the experience of Monowara (Respondent 7), a 35-year-old with secondary school completion, embroidery entrepreneur and recipient of the Joyita Award in 2016, who stated:

*I had done some embroidery at home before, but the handicraft training really helped me refine my skills. I could understand the designs and instructions better because I studied up to secondary school. That made the training more useful for me than for some other women who hadn’t gone to school. The technical training, though, was a bit too advanced and not that relevant to the kind of embroidery work I do. But overall, the training helped me improve my products and sell more. I think having some schooling helped me benefit more from the practical parts of the training.*

Education at the high school level or higher in this sector rendered training programs even more effective. The synergistic effects were particularly strong for market-oriented training (+3,214 vs. 0) and handicraft skills training (+1,066 vs. 0), where more educated entrepreneurs obtained larger improvements compared to those with secondary or primary education. On the other hand, management-level training (+2,016 vs. +2,016) and communication and negotiation training (+1,444 vs. +1,444) produced equal effects across education levels. A notable trade-off was observed in technical knowledge training, which was less effective for entrepreneurs with higher education (+1,829 vs. +2,387), suggesting that such training may have been more relevant for those with lower educational attainment.

This result resonates with the qualitative account of Parvin Aktar (Respondent 3), a 32-year-old high school educated embroidery entrepreneur and recipient of the NGO Fair Prize in 2016. She explained:

*Because I studied up to high school, I found the market-oriented and handicraft training very effective. I learned how to plan better, deal with customers, and even explore new markets. The management and communication training also helped me set fair prices and promote my products. However, the technical knowledge training did not add much value in fact, some parts felt like a step back. I already had those basic skills, so I did not benefit as much. I think that training was more helpful for women with less education or experience.*

Parvin’s reflections emphasize that her high school education improved the benefits she gained from market-oriented and handicraft skills development training, as well as from management and communication training, while limiting the added value of more basic technical instruction. This was because she already possessed those foundational skills, making the training feel repetitive rather than transformative. This highlights the importance of aligning training programs with participants’ educational levels to ensure that learning remains both relevant and impactful.

#### 4.4.3 Basket-weaving sector

In the basket weaving sector, women entrepreneurs benefited from training programs on management (+2,016 = +2,016 + 0), technical knowledge development (+1,678 = +1,678 + 0), managerial skills development (+1,529 = +1,529 + 0), and communication (+1,444 = +1,444 + 0). Market-oriented training, handicraft skills training, financial skills training had no significant effect, which is a similar pattern to the embroidery sector. These effect sizes indicated in the parentheses apply to those women with education below secondary school level.

With secondary school education, the effects of handicraft skills training became significantly more pronounced (+742 vs. 0) compared to those with education at a level below secondary. This tendency was thus observed across all three sectors, suggesting that secondary school education has synergistic effects with handicraft skills training, regardless of the sector. On the other hand, technical knowledge training was less effective (+866 vs. +1,678) for those with secondary school education. The effects of other training programs were not moderated by secondary school education, which is the same as for the two other sectors.

Education at the high school level or higher in the basket weaving sector was associated with greater gains from specific training programs. The effects were particularly strong for market-oriented training (+3,214 vs. 0) and handicraft skills training (+1,066 vs. 0), as observed for the embroidery sector, where more educated entrepreneurs reported larger improvements compared to those with secondary or primary education. On the other hand, management-level training (+2,016 vs. +2,016) and communication and negotiation training (+1,444 vs. +1,444) produced equal effects across education groups. An evident trade-off emerged in technical knowledge training, which was less effective for entrepreneurs with higher education (+1,120 vs. +1,678), suggesting that such training may have been more relevant for those with lower educational attainment.

Several IDIs highlighted persistent barriers that limit gains in the basket weaving sector. For instance, Shokina Begum, a 40-year-old basket weaver (Respondent 37), shared:

*"We have learned new techniques through the training, and our baskets are better than before. But despite these improvements, we often do not get fair prices. Bamboo has become expensive, and customers bargain a lot. So, even after learning new skills, my earnings have not increased as much as I hoped."*

These testimonies reveal that while skills were enhanced, seasonal demand, low recognition of weaving, and lack of raw materials constrained broader impacts. Such findings are consistent with evidence from the handloom and weaving sectors in Bangladesh, where market disadvantages and undervaluation limit income potential (Liton et al., 2016).

## 5 Discussion

This study demonstrates that the determinants of income growth among women entrepreneurs differ significantly across tailoring, embroidery, and basket-weaving micro-enterprises. The effectiveness of support services such as microcredit, training, production inputs, and market exposure depends on both sectoral contexts and women’s individual characteristics, particularly education and agency. In line with Kabeer’s empowerment framework, resources alone are insufficient; their impact is mediated by women’s ability to exercise choice and act upon opportunities (Kabeer, 2005). Although women across sectors may receive similar institutional support, outcomes diverge due to the interplay of individual capacities, sectoral dynamics, and broader structural conditions.

The findings show that short-term microcredit had little effect on income, while long-term loans were more effective, especially in tailoring, by enabling sustained investment in raw materials and production. This aligns with studies in Bangladesh showing that appropriately structured microcredit improves women’s livelihoods, particularly when connected to income-generating activities (Ahmed et al., 2021a; Ahmed et al., 2021b; Rahman and Haque, 2020).

For example, Jahan Ara, a 32-year-old tailoring entrepreneur (Respondent 25), explained:

*“I would be able to buy fabric in bulk with bigger loans. The small ones finish quickly, and then I cannot take orders properly.”*

Her case illustrates how larger loans supported steady production, advance orders, and better supplier negotiations, while giving her autonomy in decision-making. It is noted that tailoring entrepreneurs have the highest income and are relatively younger and higher educated than the other two groups of entrepreneurs. They are able to plan the use of bigger loans. This reflects Kabeer’s (2005) view that resources only become empowering when strategically deployed through agency. Similarly, production support services such as access to raw materials, cash donations, and stall allotments at local trade fairs had a clear positive effect on women’s income. These services directly reduced production costs and created opportunities to showcase products to wider markets, thereby improving both sales and visibility. Together, they contributed not only to immediate gains but also to longer-term market recognition. By contrast, financial assistance to attend trade fairs had only a modest impact. While it helped cover travel or participation costs, it did not overcome the more critical barriers of product display, customer access, or competitive pricing. Without stalls or direct opportunities to connect with buyers, women could not fully capitalize on these events, making this form of support less transformative than material assistance or stall provision.

This was reinforced by the experience of Lubna Afaj (Respondent 21), a 35-year-old basket weaver, who explained:

*“When I received raw materials support, it reduced my costs and I could produce more baskets without worrying about borrowing money. Later, when I got a stall at the local trade fair, many new buyers came to see my products. Some shopkeepers even placed regular orders. Without that stall, I would not have reached those customers.”*

Lubna Afaj’s account demonstrates how production-related support supports lowered entry barriers and expanded market access, directly aligning with the regression results that showed positive effects of material provision and stall allotments.

Regarding training programs the study found, it emerged as universally important, though their effectiveness varied by sector and education.

Table 10 shows that management training, managerial skills development, technical knowledge, and communication and negotiation training proved broadly effective across tailoring, embroidery, and basket-weaving sectors for women with below secondary school level. These types of training addressed universal challenges such as organizing work, managing costs, applying technical improvements, and dealing with customers, which benefitted women regardless of sector. These findings are echoed in studies from Uganda (Namatovu et al., 2019) and Ethiopia (Abebe and Mitiku, 2018). By contrast, handicraft and market-oriented training showed more selective effects, working best for women with higher education, while financial training had little or even negative impact.

**Table 10:** Training effectiveness by sector.

Training type	Tailoring	Embroidery	Basket weaving
Management development Training	Effective (esp. for less educated)	Effective (all levels)	Effective (all levels)
Managerial skills development	Very effective (largest impact on less educated)	Effective	Effective
Technical knowledge	Effective (esp. for less educated; less impact for highly educated)	Effective (strongest for less educated)	Effective (esp. for less educated)
Communication and negotiation	Effective	Effective	Effective
Handicraft skills training	Effective (amplified by secondary/higher education)	Limited effect unless combined with higher education	Effective (with secondary/higher education)
Market-oriented training	Not significant overall; but effective with higher education	Effective only with higher education	Effective only with higher education
Financial skills training	Negative effect	No significant effect	No significant effect

Source: Authors’ field survey in January to June 2017.

In the tailoring sector higher education amplified the benefits of handicraft and market-oriented training, showing synergies between education and skills. Literacy and numeracy enabled them to follow complex instructions, adapt new designs, and apply advanced techniques. Market-Oriented training also proved effective only for higher-educated women, as it required comprehension of pricing strategies, branding, and customer analysis, as well as the confidence to interact with buyers beyond local markets. Communication and negotiation training, however, was useful for both groups: less educated women applied it in daily bargaining with local customers, while more educated women used it to engage with institutional buyers and larger orders. Interestingly, financial skill development training negatively affected income in this sector, suggesting a mismatch between training content and tailoring business realities. Similar patterns are noted elsewhere, where market-oriented and handicraft skill development training improved women’s microenterprise outcomes, while financial literacy programs were less effective when not tailored to context (Rahman, 2019; Ferdousi and Mahmud, 2019).

In the embroidery sector, income growth was strongly linked to technical skills development training followed by management training for women with lower education. Since technical knowledge training provided practical skills, they could immediately apply. However, market-oriented and handicraft skill development training only became significant when combined with higher education, suggesting that literacy and comprehension were crucial for women to fully benefit. Women with high school education or higher showed greater improvements in marketing and product design. This reflects the role of education as a gatekeeper: only better-educated embroiderers could fully benefit from branding and marketing-related training. Interestingly, embroidery entrepreneurs are often positioned between tailoring and basket- weaving their income is the lowest of the three groups, and scaling up production seems more difficult. It was found that technical, communication and negotiation training were often delivered by NGOs at the local level, while government programs also provided a range of managerial and sector-specific training. The outcomes, therefore, reflect not only the content of the training but also how well it matched the needs of women in different sectors. In particular, localized and more frequent training with closer follow-up appeared especially useful for older and less-educated women. Taken together, NGO and government initiatives played complementary roles in enhancing women’s skills, though their impact varied according to education, follow-up, and sectoral context. Previous studies also highlight the importance of these dynamics: Alam et al. (2019) and Ferdousi (2020) note that literacy enhances women’s ability to absorb entrepreneurial training, while for more educated women, basic technical training often yields diminishing returns due to redundancy.

Basket-weaving showed a similar pattern: management, technical knowledge, and communication and negotiation training improved income, while handicraft skill development training was effective for women with secondary education. At higher education levels, both market-oriented and handicraft skill development training became highly beneficial, while technical training again showed diminishing returns. It is noted that technical training and communication training were offered more by NGOs, who applied aa more localized training. Since basket-weaving entrepreneurs are relatively older and less educated, they might have found the localized NGOs’ approach more beneficial.

Both government and NGO programs provided training to rural women entrepreneurs, though their approaches differed. NGOs typically organized technical, communication, and negotiation training at the local level and often followed up with participants afterwards. This close support was especially valued by older and less-educated women, who reported that such follow-up made it easier to apply new skills in practice. By contrast, government offices delivered sector-specific training such as management level training, market-oriented, managerial development, handicraft skill development and financial management training through more formal sessions at their offices. These sessions reached larger groups but involved less individual monitoring. Regression results reflected these patterns: technical and communication training (commonly NGO-led) were widely effective, while managerial and sector-specific training (often government-led) showed stronger effects among women with higher education. Thus, while both providers contributed to women’s skill development, their training was perceived differently: NGO training as practical and supportive, and government training as structured and more suitable for educated participants.

These sectoral variations illustrate that education while not directly increasing income served as a critical resource that amplified the impact of certain types of training, particularly those requiring literacy and comprehension (e.g., handicraft and market-oriented training). Conversely, technical training appeared to yield lower returns among highly educated women, suggesting that training must be aligned with participants’ prior knowledge and agency-enhancing structures. This aligns with Kabeer’s empowerment framework, which emphasizes that resources such as training and education only translate into transformative achievements when exercised through agency and supported by enabling contexts (Kabeer, 1999).

Overall, sectoral differences in income growth reflect both economic realities and gendered constraints, consistent with prior evidence of women’s enterprise performance in South Asia (Ahmed et al., 2021a; Ahmed et al., 2021b; Rahman and Haque, 2020). Tailoring, driven by standardized skills and steady local demand, responded strongly to managerial and communication training, such as order management, pricing, and customer relations, which enabled women to handle larger orders and negotiate effectively. By contrast, formal financial training had little effect, as its abstract content in areas like accounting or bookkeeping was poorly matched to the immediate realities of small, home-based tailoring businesses.

Embroidery, which relies heavily on creativity and design innovation, showed the strongest gains from technical training, improving both product quality and product differentiation. For many entrepreneurs, attending training sessions, particularly those with NGO follow-up was empowering in itself, as it provided exposure, recognition, and motivation beyond the skills acquired (Alam et al., 2019; Ferdousi, 2020). These aspects enhanced their confidence and ability to innovate, reinforcing the practical value of technical training in the sector.

Basket-weaving in rural Bangladesh is highly constrained by seasonal demand that limits consistent sales, while low recognition and valuation of baskets in the market reduce income potential. Additionally, access to raw materials such as bamboo is often limited and expensive, further constraining production. These challenges are compounded by competition from cheaper alternatives and fluctuating customer preferences, making basket weaving a vulnerable and resource-dependent livelihood. Such constraints reflect broader supply-side and market-access bottlenecks that are common in the handicraft sectors of Bangladesh and other developing countries (Chowdhury and Amin, 2021; Abebe and Mitiku, 2018).

These findings reinforce Kabeer’s (2005, 2020) argument that empowerment requires not only resources but also the capacity to define and pursue goals within structural and cultural constraints, and they extend this by showing sector-contingent returns to similar inputs. Support services are most effective when tailored to the realities of each sector: tailoring benefited most from long-term credit and production inputs, in line with management- and finance-linked gains; embroidery required stronger branding and marketing, consistent with market-access constraints in Alam et al. (2019) and Ferdousi (2020); and basket-weaving needed reliable raw material access and demand stabilization, as also noted by Chowdhury and Amin (2021).

## 6 Conclusion

This study examined the effects of training and support services on income growth among women’s micro-enterprises in tailoring, embroidery, and basket weaving sectors in rural Bangladesh, using a mixed methods design with a quantitative survey of 170 entrepreneurs and 29 qualitative interviews. Normalized real income rose significantly in all sectors, with embroidery showing the largest percentage gain and tailoring the highest absolute increase. Effects were clearly differentiated: tailoring benefited most from managerial and handicraft training, alongside long-term microcredit; embroidery and basket weaving gained primarily from technical training and production-material support, with trade-fair stall allocations boosting market access. Long-term (two-year) credit and input provision had consistently positive effects, while short-term credit effects were weaker or insignificant. In all likelihood, the evidence supports tailored sector-specific packages that pair appropriate skills development with inputs and long-term finance, rather than one-size-fits-all interventions.

This study contributes to the literature on gender and micro-enterprise development in several ways. First, it provides a sector-specific perspective, revealing that uniform support services lead to different outcomes across tailoring, embroidery, and basket weaving due to sectoral characteristics and social valuation. Second, it extends Kabeer’s empowerment framework by demonstrating how access to targeted training, production inputs, and long-term credit enhances women’s agency in managing and growing their enterprises. Third, it highlights the importance of aligning support services with sector-specific needs rather than adopting one-size-fits-all approaches. Fourth, the study delivers policy-relevant insights by identifying the types of support most effective in each sector. Finally, it offers a transferable framework to analyze how institutional support interacts with barriers and women’s agency guiding the design of more context-sensitive, equitable, and sustainable programs in similar rural economies.

From a policy perspective, one-size-fits-all enterprise support is inadequate. Programs should tailor interventions to sector-specific needs, combining targeted training with long-term credit and production-material support. Training should consider participants’ education levels to ensure comprehension and effectiveness. Market access can be supported through mechanisms included in the study, such as trade-fair stall allocations. These findings emphasize the importance of designing context-sensitive, evidence-based support packages that enhance women’s micro-enterprise performance in rural Bangladesh.

This study has several limitations. First, the focus on two unions in Tangail constrains external validity. Multi-district nationally representative panels should be employed to test whether sectoral patterns are generalized. Second, the 2014 outcomes rely on self-reported recall data. Future research should triangulate it with administrative records, sales ledgers, mobile-money data, and sector-specific price indices. Third, the time lag between data collection (i.e., 2017) and the present publication year (i.e., 2025) may affect the current applicability of some findings. Fourth, the observational design cannot rule out self-selection into training, credit, and trade fairs. In addition, non-response bias may be present. Phased roll-outs, eligibility thresholds, randomized encouragement, or strong instruments (e.g., distance to providers) are needed for better causal identification. Fifth, small subsample sizes, especially in basket weaving, may have reduced statistical power. Larger cohorts and parsimonious pre-registered models with key interactions would improve precision and help test mechanisms. Lastly, binary treatment measurements obscure heterogeneity. Collecting intensity and quality (e.g., hours, duration, curricula, loan size, loan terms, number of fairs) and using panel data models would help understand the dynamics better.

## Data Availability

The data analyzed in this study is subject to the following licenses/restrictions: the data supporting the findings of this study were collected from rural areas of Bangladesh and contain sensitive information related to participants. Therefore, the data are not publicly available due to ethical and privacy considerations. Requests to access these datasets should be directed to JB, jakia_begum99@yahoo.com.

## Footnotes

^1^IMF, International Financial Statistics & Data Files: https://data.worldbank.org/indicator/FP.CPI.TOTL.ZG?locations=BD

^2^USD = 77.6 BDT in 2014 & 81.2 BDT in 2017.
